# The Compulsory Care Act: Early Observations and Expectations of In- or Outpatient Involuntary Treatment

**DOI:** 10.3389/fpsyt.2021.770934

**Published:** 2022-02-08

**Authors:** Stephan Gemsa, Eric O. Noorthoorn, Peter Lepping, Hein A. de Haan, Andre I. Wierdsma, Giel J. M. Hutschemaekers

**Affiliations:** ^1^Ggnet Mental Health Institute, Child Psychiatry Service, Warnsveld, Netherlands; ^2^Betsi Cadwaladr University Health Board, Wrexham, United Kingdom; ^3^Wrexham Academic Unit, Centre for Mental Health and Society, Bangor University, Wrexham, United Kingdom; ^4^Mysore Medical College and Research Institute, Mysuru, India; ^5^Tactus Verslavingszorg, Addiction Care and Treatment Service, Deventer, Netherlands; ^6^Department of Psychiatry, Erasmus Medical Centre, Rotterdam, Netherlands; ^7^Behavioral Science Institute, University of Nijmegen, Nijmegen, Netherlands; ^8^Pro Persona Mental Health Care, Indigo Centre, Nijmegen, Netherlands

**Keywords:** compulsory care act, coercion, seclusion, enforced medication, community treatment order, involuntary inpatient treatment, involuntary outpatient treatment

## Abstract

**Background:**

On January 1, 2020, the Dutch Compulsory Care Act (WvGGZ) replaced the Special Admissions Act (BOPZ). While the old law only allowed compulsory treatment in hospitals, the new law allows it both inside and outside the hospital. Moreover, the new law prioritizes the patient's own opinion on coercive measures. By following patients' own choices, the Compulsory Care Act is hoped to lead to fewer admission days and less inpatient compulsory treatment in involuntarily admitted patients.

**Methods:**

We studied the seclusion and enforced-medication events before and after January 1, 2020, using coercive measures monitoring data in a Mental Health Trust. Trends in hours of seclusion and the number of enforced-medication events per month from 2012 to 2019 were compared with 2020. We used generalized linear models to perform time series analysis. Logistic regression analyses and generalized linear models were performed to investigate whether patient compilation determined some of the observed changes in seclusion use or enforced-medication events.

**Results:**

The mean number of hours of seclusion between 2012 and 2019 was 27,124 per year, decreasing from 48,542 in 2012 to 21,133 in 2019 to 3,844 h in 2020. The mean incidence of enforced-medication events between 2012 and 2019 was 167, increasing from 90 in 2012 to 361 in 2019 and then fell to 294 in 2020. In 2020, we observed 3,844 h of seclusion and 294 enforced-medication events. Near to no outpatient coercion was reported, even though it was warranted. The time series analysis showed a significant effect of the year 2020 on seclusion hours (β = −1.867; Exp(β) = 0.155, Wald = 27.22, p = 0.001), but not on enforced-medication events [β = 0.48; Exp(β) = 1.616, Wald = 2.33, *p* = 0.13].

**Discussion:**

There was a reduction in the number of seclusion hours after the introduction of the Compulsory Care Act. The number of enforced-medication events also increased from a very low baseline, but from 2017 onwards. To see whether these findings are consistent over time, they need to be replicated in the near future.

**Conclusion:**

We observed a significant increase in enforced-medication use and a decrease in seclusion hours. The year 2020 predicted seclusion hours, but not enforced-medication events.

## Introduction

On January 1, 2020, the Dutch Compulsory Care Act (WvGGZ) (2020) (1) replaced the Special Admissions Act (1994) (BOPZ) (2). The BOPZ was primarily designed to regulate compulsory admissions, but not treatment. The Act was evaluated in 1997, 2002, and 2007. After the second evaluation, conditional authorization was introduced (3). This allowed the possibility of outpatient treatment with conditions. This may be seen as outpatient persuasion under duress, in effect coercion in an “or else” formulation (4). The aim was that patients could be discharged more quickly and that, if possible, inpatient treatment would not be necessary if patients could comply with the conditions. The main condition was usually to adhere to treatment policy and take the prescribed medication. Furthermore, the second evaluation concluded that the law was too much focused on patients' rights and too little on treatment. As a response, legislators developed the Compulsory Care Act. This legislation focuses on treatment rather than admission. While the Special Admissions Act only allowed compulsory treatment in emergency situations in hospitals, the new act allows compulsory treatment in both inpatient and outpatient settings. The conditions for compulsory outpatient treatment are authorized by a judge in a community treatment order (CTO). Outpatient involuntary treatment may include enforced medication, supervisory measures, and admission as the ultimate remedy. An important motivation for the new law was the assumption that a CTO will lead to fewer admission days and fewer inpatient coercive measures such as seclusion or enforced medication in patients who are involuntarily admitted (3, 5).

In summary, the new Compulsory Care Act regulates the provision of mandatory care for people with severe mental illness. Mandatory care is precisely described in a care plan authorized by a judge. It focuses on outpatient care supplemented with optional inpatient care, which by law has to prevent serious disadvantages for the patient.

The Compulsory Care Act maintains the same principles of subsidiarity, proportionality, and expediency as described in the Special Admissions Act (6):

- Subsidiarity: a more intrusive measure is only allowed when a lesser intrusive measure is insufficient to prevent danger.
- Proportionality: the measure needs to be proportionate to the extent of the danger. The infringement on autonomy or bodily integrity should not exceed the danger that the patients may pose to others or themselves. The safety of the measure should be weighed against the risks if no action is taken. The psychiatrist or the authorized therapist must document which efforts were taken to ensure patients' rights.
- Expediency: the treatment or measure must have proven efficacy in dealing with the danger that the patients pose.

Evaluations of the Special Admissions Act (3) pointed out that it would not comply with principles of the Convention on the Rights of Persons with Disabilities (CRPD) (7–9). The new legislation has therefore been developed from a patient perspective in close collaboration with the relevant patient associations. The experience of patients and that of their next of kin were considered in the design of procedures. Social participation, preservation of as much personal autonomy as possible, and focus on treatment with as little coercion as possible are the basic principles of the new legislation. When the Special Admissions Act was in place, seclusion was the coercive measure of choice (87% of nationwide coercive measures) (10). When patients were asked about their preference, a majority preferred medication over seclusion (11). In the new law, at the start of any involuntary treatment, a judge includes the patient's own opinion in the choice of measure. Consequently, enforced medication may now be expected to be used more often than seclusion (5, 12).

Before the introduction of the Special Admissions Act in 1994, registration of separate coercive measures was not regulated (9). Only seclusion and mechanical restraint, but not enforced medication, were identified as coercive measures. Measures occurring within 2 h did not need to be reported to the Mental Health Inspectorate. Measures above 2 h were reported, often in retrospect a number of days after the event occurred. In several publications, the accuracy of these data is questioned (13). After the introduction of the Special Admissions Act, it became mandatory to report all coercive measures to the Inspectorate. The Special Admissions Act clearly defined coercive measures as seclusion in high- and low-security rooms as well as the patient's own room, mechanical and physical restraint, forced medication, forced fluids and forced feeding, and very rarely electroconvulsive therapy (ECT) when given against the patient's will (13). These measures were recorded according to their legal validity period rather than their actual duration (6). This led to an overestimation of time in seclusion and an underestimation of the number of times that enforced medication, forced fluids, and forced feeding were used between 1994 and 2006 (14, 15).

Even though the Special Admissions Act was primarily a law regulating involuntary admissions (3), it did allow coercive measures as a last resort. In Dutch daily psychiatric practice, however, any breach of the integrity of the body by means of enforced medication was interpreted as a higher degree infringement of the patient's human rights than seclusion. This was an interpretation not based on patients' opinions (11). As an effect of the absence of effective treatment, seclusion duration was much higher than in other European countries (10, 16).

In 2004, the Dutch mental health organization, GGZ Nederland, formulated a policy statement detailing that psychiatric hospitals should reduce seclusion at a rate of 10% per year (15). In 3 rounds of Governmental funding, 35 million Euros were invested in 55 seclusion-reduction programs (15). Several best-practice protocols were developed (12), a number of which were evidence based and a number of which were practice based. These protocols were designed to change ward culture. All of these practices were aimed at engaging the patient (12). In addition, the hospital environment was adjusted, including single-person bedrooms, comfort rooms, family rooms, and low-threshold access to nurses in the ward or behind accessible counters, rather than in nurse stations. All these changes were evidence based and aimed at improving the ward environment. These programs were started in 2006 (15) and intensified after 2012 (17).

From 2006 up to 2012, an increasing number of Dutch psychiatric hospitals engaged in the voluntarily monitoring of their own data as part of the nationwide seclusion-reduction program. Data were analyzed in anonymous databases at the level of coercive measures and patient admissions (10). In 2010, half of the large Mental Health Trusts participated. In 2012, the Argus coercive measures (13) rating scale was included in the BOPZ legislation. Between 2012 and 2014, nationwide data were gathered. In 2014, all Trusts participated. Data gathered in the nationwide databases (10, 12, 16) and through open sources (18) showed that the seclusion-reduction programs led to a sharp decrease in seclusion use in some but not all hospitals. Overall, the decrease was more evident in the first 5 years of the reduction programs but then plateaued (18, 19). Recent findings from some hospitals show that the sharp reduction in seclusion hours is possibly related to the increased use of enforced medication (6, 20, 21). Cross-sectional data gathered in 2014 showed an association of seclusion time reduction with the development of high and intensive care units (17). However, despite the large investment in seclusion-reduction programs and in designing and building intensive care wards following the UK and Scandinavian examples, the nationwide results remained disappointing. Nationwide findings after 2012 showed that an initial reduction of seclusion hours between 2012 and 2016 was followed by an increase between 2017 and 2018 (18). The large differences in trends between Mental Health Trusts observed between 2006 and 2012 consolidated later on, showing that some Mental Health Trusts had 10 times higher seclusion use rates than others (12, 18). A possible explanation may be that many hospitals only partly included best practices and high and intensive care (21, 22).

It has been well-established that coercive measures are traumatizing when applied and should be avoided whenever possible. Both measures, seclusion and enforced medication, are experienced as severely traumatizing by patients (23). Coercive measures cause trauma for both patients (24) and nurses (25). In daily practice, carrying out coercive measures is time-consuming and impairs nurses in providing adequate care. It disturbs building a therapeutic relationship. Nurses are engaged in containing behavior rather than in coming into contact (26). The high and intensive care policy that was developed in 2012 aimed to reduce coercive measures as much as possible in keeping with these findings (16, 21).

When the Compulsory Care Act (2020) was introduced, the legislator's expectation was that the focus of psychiatric treatment be on outpatient treatment at an earlier stage, with coercion, if necessary, in order to result in fewer admissions and less inpatient compulsory treatment (5, 8). Table 1 depicts the main differences between both laws.

**Table 1:** Differences between both laws.

**Special admissions act (BOPZ)**	**Compulsory care act (WvGGZ)**
Focus on admission	Focus on treatment
Inpatient involuntary treatment (coercion)	In- and outpatient involuntary treatment (coercion)
5 different coercive measures possible	11 different coercive measures possible
6 different types of authorization	3 different types of authorization
Outpatient conditional treatment	Outpatient involuntary treatment
No direct family participation	Active family participation
No direct or active patient participation	Patient involvement required (own plan of action)
Danger criterion as a legal requirement for coercion. Proportionality, subsidiarity, efficiency and safety checked by the Mental Health Inspectorate	Proportionality, subsidiarity, efficiency and safety as legal requirements for coercion. Coercion authorized by a judge

The current study investigates the effect of the conceptual change in the law by examining whether changes in seclusion and enforced-medication use have indeed occurred. We expect coercive measures to be more in line with the patients' own choice. We expect a decrease in seclusion and an increase in the number of medication events.

## Methods

### Materials

The data were gathered from a large Mental Health Trust at the east of the Netherlands, with a catchment area of just above 600,000 inhabitants (27). In the Dutch context, this is a medium size trust with a semi-rural population associated with a lower prevalence of involuntarily treated patients (11). The eligible population at risk of coercive treatment includes all involuntarily treated patients, and this covers inpatients and outpatients. This concerns approximately 5% of all psychiatrically admitted patients in a large European sample (15); in our study example, it is estimated at ~300 patients a year, which was a reasonably constant figure in our database. Data on coercive measures were mandatory and gathered for the Mental Health Inspectorate. For the purpose of this and previous studies, the data were fully anonymized. One consequence of this anonymization is that we do not know whether patients admitted in 1 year were readmitted in another.

Before the implementation of the new law, the Argus coercive measures (14) rating scale was fully integrated into the data collection. The Argus coercive measures rating scale includes items such as seclusion, restraint, involuntary medication, forced administration of fluids and nutrition, and miscellaneous, extremely rarely used interventions such as ECT or intravenous medication. With every actual application of one of these interventions, a date, start time, and end time are noted (no end time in the case of involuntary medication). This is further complemented by documentation of the observed degree of patient resistance to the intervention. In the analysis, the use of coercive measures per patient was used as counters and the number of involuntarily treated patients as denominators. This is done to standardize the findings and to calculate trends over time, independent of organizational changes (9, 11, 14).

Seclusion is defined in the Argus set as follows: locking a patient in a specially designated and Dutch Mental Health Inspectorate-approved room for the purpose of care, nursing, and treatment. Involuntary medication or chemical restraint is defined as intramuscular intervention medication given to the patient under clear visible and notable resistance. As a denominator, it contains admission and discharge date. Patient characteristics such as age, gender, diagnosis, and ward type are included as modifying or confounding variables (14).

After the implementation of the Compulsory Care Act, a compulsory care database was introduced. It uses partly the same items as the Argus dataset but introduces a number of new items. We only present the comparable items from the two databases in the current publication.

### Data Organization

For the purpose of the analysis, three databases were constructed. The first contained the counters, i.e., the coercive measures, either within the BOPZ or within the WVGGZ. It contained each measure with the start and end times of each episode. The second contained patient background data such as admission date, discharge date, date in and out of outpatient care, age, and diagnosis. The third contained information on legal status including the start and end times of each legal measure. With these three databases, all trends presented could be calculated. Between the several databases, checks on primary and secondary keys are done to deselect errors such as double records, inappropriate duration data, and inappropriate patient allocations to wards. Primary keys concern the lowest level, i.e., the data of the coercive measures. Secondary keys concern the patient background data at admission or outpatient treatment level. When a patient is allocated to a ward in one source, the patient needs to be allocated to the same ward in another source. Detected differences were corrected by research nurses.

To allow a time series analysis, the first database of coercive measures was aggregated to 108 months: 96 before and 12 after the implementation of the new law. To allow an investigation of patient characteristics as confounders of the main outcome measures, seclusion, and enforced IM medication, the first database of the separate coercive measures was aggregated to the number of seclusion events and seclusion hours per patient per year. In addition, we aggregated the number of medication events per patient per year. This was merged into the admission data, covering age, gender, year of admission, and diagnosis. Over the 9 years from 2012 to 2020, a single database was constructed and added to a previous year. Anonymization of the data necessitated that we did not know which patients may have been included again in the data of a further year.

### Statistical Procedures

#### Basic Frequencies

We present findings from 2012 to 2020 in five trend figures. The first figure covers the number of seclusion incidents as defined by Janssen et al. (14). In this definition, a seclusion incident can be defined as a number of discrete episodes following each other. An incident is derived from the epidemiological term incidence and can cover a sequence of episodes without discontinuation for more than 24 h. An interruption of more than 24 h leads to the count of a new incident of seclusion. Enforced-medication incidents are always counted as single episodes. **Figure 2** presents the percentage of patients subjected to seclusion and enforced-medication incidents. This figure is well-comparable with such figures internationally (12, 15).

#### Time Series Analyses

Time series analyses were performed including the 108 months between 2012 and 2020 to evaluate the effect of the new legislation on the use of seclusion and involuntary medication. Each record contained an identifier for the month, the season, the number of seclusion hours, and the number of involuntary medication events. A time series analysis is an option in the generalized linear models of SPSS software. To model changes, we used included autocorrelation, linear trend and seasonal effects, and an indicator for the introduction of the new legislation (28–31). The number of seclusion hours was analyzed using a quasi-Poisson generalized linear model, as this deals with slightly skewed counts (skewness, 0.34; kurtosis, 0.55). The number of medication events was analyzed using generalized linear models with negative binomial log link function, as these deal with highly skewed counts (skewness, 2.39; kurtosis, 7.84). Model selection was based on the Wald tests with alpha set at 5%, using SPSS (version 27).

#### Regression of Seclusion and Enforced Medication by Age, Gender, Year, and Diagnosis

We performed a logistic regression analysis and *post-hoc* generalized linear models with negative binomial log link because we identified a significant trend in the time series analysis (29). Generalized linear models are needed to explore the underlying variables that may explain the trend. Generalized linear models with negative binomial link are specifically designed for skewed variables with many zeros. In our seclusion data, this is the case in 1,918 out of 2,838 records; in the medication data, it is the case in 2,304 out of 2,838 records.

We did a logistic regression to exclude that the influence of patient compilation did not by chance explain the trend. Logistic regression of having been secluded and having received enforced medication by age, gender, diagnosis, and year was done and corrected for case mix. Case mix analysis looks into whether patient compilation in a certain year has an effect on the chance of being secluded or receiving enforced medication. We first analyzed patient compilation over time by performing a crosstabulation of patient characteristics and having been secluded or having received enforced medication per patient per year. We then performed a logistic regression analysis to investigate whether patient compilation was associated with less or more chance to be secluded or receive enforced medication. After that, we added the generalized linear models. To allow a better interpretation of both regression analyses, we constructed dummy variables for age categories, diagnosis on axis 1, and diagnosis on axis 2. In the tables, the reference categories are presented in brackets. We presented the findings in keeping with the suggestions of the American Statistical Association (32).

## Results

**Figure 1:**
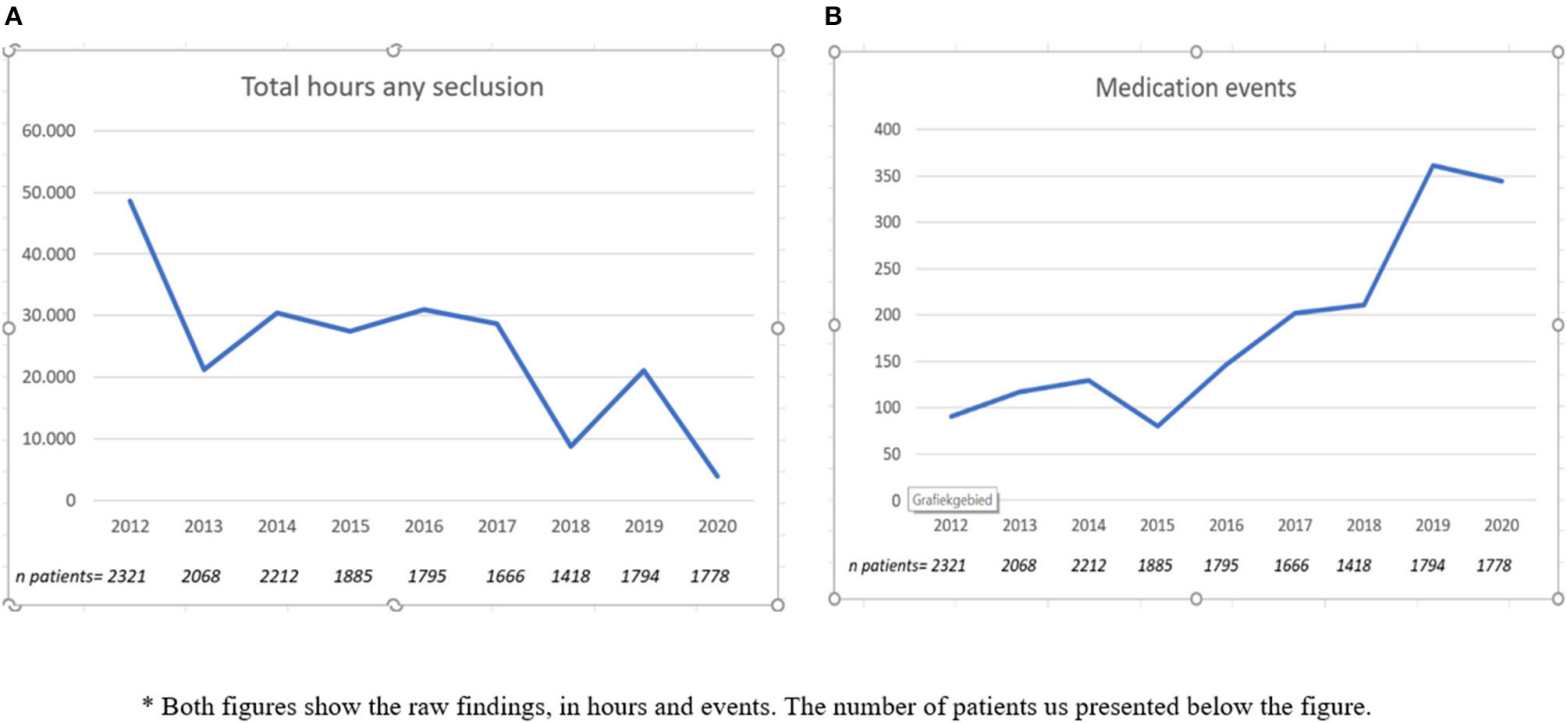
Trends in seclusion hours and medication events*. *Both figures show the raw findings, in hours and events. The number of patients us presented in the figure.

The patient-level database contained 13,162 records at one record per patient per year. The patient-level database that included only involuntarily admitted patients contained 2,838 records, again with one record per patient per year. The trend data contained 108 records, one record per number of seclusion hours or medication events per month. The first finding of interest was the number of hours of seclusion and medication events over time. Figure 1A presents the seclusion hours, whereas Figure 1B shows the medication events. A clear decrease in seclusion hours against a rise in medication events could be seen. While in 2012 we counted 48,542 h of seclusion, this figure dropped to 3,844 in 2020, a 92% decrease. In more detail, between 2012 and 2014, a clear decrease from 48,542 to 30,398 h could be observed. Between 2014 and 2017, seclusion hours stabilized at ~30,000 h to decrease again thereafter. Most of the decrease occurred in 2020, where the number of seclusion hours dropped from 21,133 to 3,844 h, an 82% decrease. The frequency of involuntary intramuscular medication increased from a very low baseline of 90 in 2012 to 361 in 2019 (301% increase) and dropped to 294 in 2020 (18% decrease). We noted that in 2020, only 8 out of the 294 medication events occurred outside the hospital environment. Outpatient coercion authorized by a CTO was therefore very rare.

The time series analysis on the data underlying these two trends showed a decrease of seclusion hours over time [β = −0.013; Exp(β) = 0.987, 95% CI Exp(β) = 0.984–0.990, Wald = 67.63, *p* = 0.001]. Second, a significant effect on seclusion hours was observed since implementation of the new law [β = −1.87; Exp(β) = 0.155, 95% CI Exp(β) = 0.077–0.312, Wald = 27.22, *p* < 0.001]. Concerning involuntary medication events, an increase over time was observed [β = 0.013; Exp(β) = 1.013, 95% CI Exp(β) = 1.006–1.012, Wald = 13.27, *p* < 0.001], but no significant effect since implementation of the new law could be detected [β = 0.48; Exp(β) = 1.616, 95% CI Exp(β) = 0.872–2.994, Wald = 2.32, *p* = 0.13]. The season showed no effect on seclusion hours or medication events.

**Figure 2:**
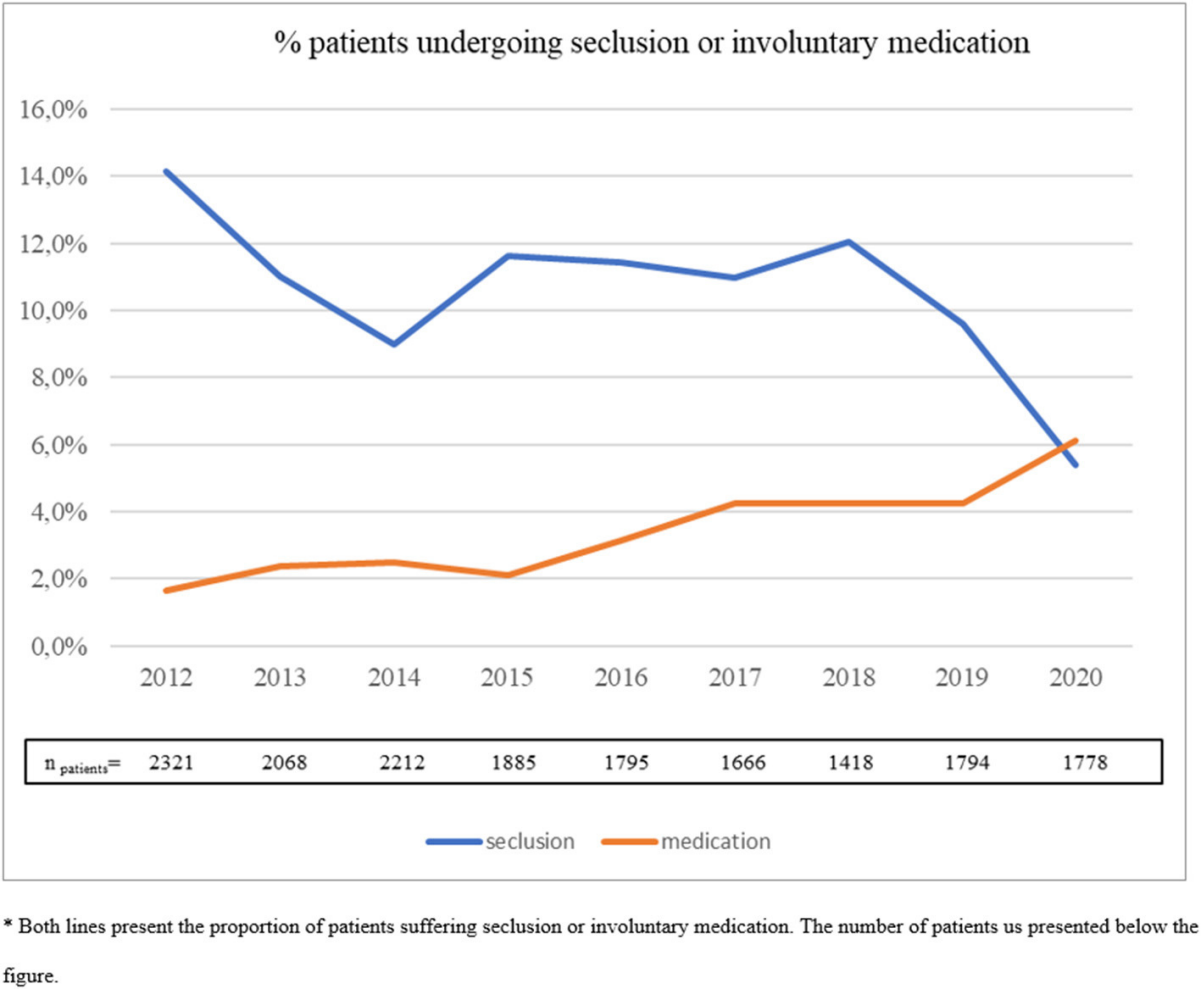
Percentage patients undergoing seclusion or involuntary medication. *Both lines present the proportion of patients suffering seclusion or involuntary medication. The number of patients us presented in the figure.

Figure 2 presents the *percentage of patients* subjected to coercive measures. We observed a clear decrease in the proportion of patients subjected to seclusion, especially after 2018. In 2020, the percentage of patients subjected to seclusion dropped, while it increased for enforced medication, and the trends crossed each other at 6%. We observed an increase in the proportion of patients undergoing involuntary medication during the time frame we investigated, especially after 2017. In 2020, the percentage of patients subjected to involuntary medication increased. The year 2020 was associated with an increase in enforced-medication events when compared with all of the years before 2020 with the exception of 2019 [Exp(β) = 2.0].

The logistic regression (Table 2) showed male gender (Exp(β) = 1.24), young (Exp(β) = 2.57) and middle age (Exp(β) = 2.17), a bipolar disorder (Exp(β) = 2.26), a psychotic disorder (Exp(β) = 1.58), and a mental handicap (Exp(β) = 1.3) predicted a higher risk of being secluded. The year 2020, when the new law was implemented, was associated with less risk of being secluded (Exp(β) = 0.41). The same analysis showed that male gender (Exp(β) = 0.78) and drug abuse disorder (Exp(β) = 0.67) were associated with a lower risk of receiving enforced medication. A psychotic disorder (Exp(β) = 1.75) was associated with an increased risk of receiving enforced medication.

**Table 2:** Logistic regression findings.

**Secluded**	**Predictor (reference)**	**Beta**	**SE**	**Exp (β)**	**95% CI Exp (β)**	
	Male (female)	0.22	0.09	1.24	1.05	1.47
	Age (older)					
	Young aged	0.94	0.15	2.57	1.92	3.44
	middle aged	0.77	0.14	2.17	1.64	2.88
	Law (before)					
	after law	−0.89	0.15	0.41	0.30	0.55
	Axis 1 Diagnose (neurotic)					
	Neurotic disorder					
	Bipolar disorder	0.82	0.16	2.26	1.64	3.10
	Autism	−0.03	0.22	0.97	0.63	1.49
	Psychotic disorder	0.46	0.14	1.58	1.21	2.06
	Schizophrenia	0.01	0.13	1.01	0.77	1.31
	Organic disorder	0.13	0.24	1.13	0.71	1.82
	Co-morbid drug disorder	0.07	0.13	1.07	0.83	1.39
	Axis 2 Diagnosis (none)					
	Personality disorder	0.24	0.12	1.27	0.99	1.61
	Mental handicap	0.32	0.15	1.38	1.02	1.39
	Constant	−1.75	0.17	0.173	
Medicated	Male (female)	−0.25	0.11	0.78	0.63	0.97
	Age (older)					
	Young aged	0.24	0.17	1.27	0.91	1.79
	middle aged	0.13	0.16	1.13	0.82	1.56
	Law (before)					
	after law	0.76	0.14	2.15	1.62	2.84
	Axis 1 Diagnose (neurotic)					
	Neurotic disorder					
	Bipolar disorder	0.35	0.20	1.42	0.95	2.11
	Autism	−0.25	0.29	0.78	0.44	1.39
	Psychotic disorder	0.56	0.17	1.75	1.26	2.42
	Schizophrenia	0.04	0.17	1.04	0.75	1.44
	Organic disorder	−0.07	0.31	0.94	0.52	1.70
	Co-morbid drug disorder	−0.38	0.19	0.67	0.47	0.99
	Axis 2 Diagnosis (none)					
	Personality disorder	−0.21	0.17	0.81	0.58	1.12
	Mental handicap	−0.25	0.22	0.78	0.50	1.21
	Constant	−1.88	0.19	0.15	

*The significant findings can be identified by their confidence interval, with no 1 included. Concerning secluded these are male, young and middle aged, the law, a bipolar disorder, a psychotic disorder and a mental handicap. Concerning medicated these are male, the law, psychotic disorder and a co-morbid drug disorder. The Exp (β) of a logistic regression may also be interpreted as an odds ratio*.

The generalized linear models with negative binomial link (Table 3) showed male gender [Exp(β) = 1.89], a younger age [Exp(β) = 4.41], middle age [Exp(β) = 2.90], personality disorder [Exp(β) = 1.52], and a mental handicap [Exp(β) = 2.17] were associated with more seclusion hours. A psychotic disorder [Exp(β) = 0.69], schizophrenia [Exp(β) = 0.70], an organic disorder [Exp(β) = 0.51], a drug abuse disorder [Exp(β) = 0.46], and the year the law was implemented [Exp(β) = 0.25] were associated with a lower chance to be secluded. The generalized linear model with negative binomial link on medication events showed a young age [Exp(β) = 1.30], a bipolar disorder [Exp(β) = 1.72], and the year the law [Exp(β) = 2.00] was implemented were associated with more medication events. Male gender [Exp(β) = 0.75], schizophrenia [Exp(β) = 0.59], comorbid drug abuse [Exp(β) = 0.35], and mental handicap [Exp(β) = 0.53] were associated with fewer medication events.

**Table 3:** Generalized linear models with negative binomial link findings.

**Seclusion hours**	**Predictor (reference)**	**Beta**	**SE**	**Exp (β)**	**95 % CI Exp (β)**	
	Male (female)	0.63	0.11	1.89	1.50	2.37
	Age (older)					
	Young aged	1.48	0.16	4.41	3.21	6.06
	Middle aged	1.06	0.15	2.90	2.14	3.93
	Law (before)					
	after law	−1.36	0.16	0.25	0.19	0.35
	Axis 1 Diagnose (neurotic disorder)					
	Bipolar disorder	0.22	0.20	1.25	0.84	1.86
	Autism	0.04	0.27	1.04	0.62	1.75
	Psychotic disorder	−0.37	0.16	0.69	0.50	0.95
	Schizophrenia	−0.35	0.16	0.70	0.51	0.96
	Organic disorder	−0.67	0.25	0.51	0.31	0.85
	Co-morbid drug disorder	−0.77	0.17	0.46	0.33	0.65
	Axis 2 Diagnosis (none)					
	Personality disorder	0.42	0.16	1.52	1.12	2.06
	Mental handicap	0.77	0.19	2.17	1.50	3.15
	Intercept	2.47	0.17	11.85	8.55	16.43
Medication events	Male (female)	−0.29	0.18	0.75	0.64	0.87
	Age (older)					
	Young aged	0.26	0.11	1.30	1.03	1.64
	Middle aged	−0.22	0.11	0.80	0.64	1.01
	Law (before)					
	After law	0.69	0.19	2.00	1.65	2.45
	Axis 1 Diagnose (neurotic)					
	Bipolar disorder	0.54	0.13	1.72	1.33	2.22
	Autism	−0.09	0.17	0.92	0.65	1.30
	Psychotic disorder	−0.13	0.12	0.99	0.79	1.24
	Schizophrenia	−0.54	0.12	0.59	0.47	0.75
	Organic disorder	−0.17	0.20	0.85	0.57	1.27
	Co – morbid drug disorder	−1.07	0.14	0.35	0.25	0.46
	Axis 2 Diagnosis (none)					
	Personality disorder	0.17	0.10	1.19	0.96	1.47
	Mental handicap	−0.63	0.17	0.53	0.38	0.74
	Intercept	−0.50	0.13	0.61	0.47	0.78

*The significant findings can be identified by their confidence interval, with no 1 included. Concerning seclusion hours these are male gender, young and middle aged, the law, a psychotic disorder, schizophrenia, an organic disorder, a co-morbid drug disorder, personality disorder and mental handicap. Concerning medication events these are male gender, young aged, the law, a bipolar disorder, schizophrenia, a co-morbid drug disorder and a mental handicap. In Generalized Linear Models, Exp (β) may be interpreted as a growth or downturn factor*.

Crosstabulation (Table 4) showed that there were fewer admissions of the elderly and patients with psychotic disorders or personality disorders in 2020. However, patients with schizophrenia were admitted more. For all other variables, the number of patients admitted varied but did not explain the change in seclusion and medication rates.

**Table 4:** Patient compilation: seclusion and medication in involuntary admitted patients over years.

	**2012**	**2013**	**2014**	**2015**	**2016**	**2017**	**2018**	**2019**	**2020**
Male gender	208 (53%)	113 (50%)	205 (57%)	181 (60%)	160 (56%)	182 (57%)	150 (56%)	197 (59%)	215 (61%)
Young age	145(37%)	85(37%)	158(44.1%)	112(37%)	103(36%)	117(37%)	94(35%)	116(35%)	125(35%)
Middle aged	201(51%)	107(47%)	165 (46%)	140 (46%)	128 (45%)	137 (43%)	128(48%)	167 (50%)	166 (47%)
Anxiety or depression	98 (25%)	46 (20%)	85(24%)	62(21%)	72 (25%)	62 (20%)	62 (23%)	134(40%)	9,393 (26%)
Bipolar	41 (11%)	23 (10%)	36 (10%)	46 (15%)	29 (10%)	32 (10%)	40 (15%)	29 (9%)	30 (9%)
Psychoses	99 (25%)	63 (28%)	92 (25%)	91 (30%)	96 (33%)	90 (28%)	86 (32%)	45 (13%)	31 (9%)
Schizophrenia	135 (35%)	48 (21%)	122 (34%)	90 (30%)	69 (24%)	89 (28%)	58 (22%)	63 (19%)	155 (44%)
Autism	30 (8%)	8 (4%)	41 (12%)	27 (9%)	32 (11%)	29 (9%)	25 (9%)	50 (15%)	40 (11%)
Drug abuse disorder	77 (20%)	20 (9%)	87 (24%)	66 (22%)	52 (18%)	63 (20%)	49 (18%)	31 (9%)	62 (18%)
Personality disorder	87 (22%)	38 (17%)	85 (24%)	35 (12%)	41 (14%)	26 (8%)	17 (6%)	41 (12%)	31 (8%)
Cognitive disorder (dementia)	2- (5%)	12 (5%)	16 (5%)	23 (8%)	25 (9%)	38 (11%)	29 (11%)	16 (5%)	14 (4%)
Intellectual Disability	28 (7%)	23 (10%)	43 (12%)	40 (13%)	29 (10%)	24 (8%)	20 (8%)	22 (7%)	34 (10%)
Number of patients secluded	154 (39%)	100 (44%)	110 (31%)	121 (40%)	102 (36%)	92 (29%)	95 (36%)	98 (26%)	58 (16%)
Number of patients receiving enforced medication	47 (12%)	40 (18%)	16 (4%)	38 (12%)	45 (16%)	62 (19%)	49 (18%)	52 (16%)	84 (24%)

## Discussion

This is the first Dutch study presenting findings on coercive measures after the implementation of a major change in the Dutch Mental Health legislation. The main finding of this study is that after the implementation of the new Act in 2020, the applied coercive measures showed a substantial change. Time series analysis of seclusion and medication showed a significant decrease of seclusion hours, albeit from a very high baseline compared with that of other countries. At the same time, there was a significant increase in the use of involuntary medication, albeit from a very low baseline internationally. The decreasing trend in seclusion proves a significant effect of the law, while the increasing trend in medication did not show an effect of the law. Regarding medication, an increase was already observed in the years before the implementation of the law. Contrary to expectations, the number of outpatient coercive medications remained very low. It is not yet clear whether this is a result of registration errors or a reluctance by clinicians to use the new legislation for outpatients.

To investigate whether patient compilation determined this outcome, we performed a logistic regression on the chance to be secluded or receive involuntary medication and a generalized linear model on seclusion hours and medication events. These analyses showed that patient compilation did not predict the changes in seclusion and involuntary medication use.

The Netherlands has a history of state-sponsored seclusion reduction that started in 2006. To some extent, this is reflected in the findings presented here. However, despite some seclusion reduction between 2012 and 2019, no clear trend was shown in the examined data until 2019. We observed an indifferent trend with higher and lower figures between 2012 and 2017 and a slight decrease in 2018 and 2019. In 2020, however, we see a clear trend toward avoiding seclusion, and a continuation of an existing trend in the rising use of involuntary medication.

The drive to reduce seclusion is influenced by several factors. Theoretically, these can be divided into two main groups: political factors and professionals' opinions. Political factors are important and reflected in changes to mental health legislation. An important additional factor in line with the CRPD is the legal obligation to include the patient's perspective about choices made in involuntary treatment into any new legislation. This obligation was advocated by patients' associations (31). Financial funding streams play a role, especially in a partially government-funded health system like the one in the Netherlands, because they allow the government to set targets and priorities for healthcare systems.

Professionals' opinions are reflected in the recent changes to guidelines combined with growing insights into how patients experience coercion. In the Netherlands, an increasing acceptance of the use of medication above seclusion can be observed within clinicians' and patients' associations. However, the practice seems hard to change, and seclusion reduction has by no means been a straightforward downward trend. In clinical practice, guidelines allow considerable room for maneuver when put into practice. This freedom is reflected in large differences between Dutch healthcare providers with differences in seclusion use of up 10 times between providers, as observed in open-source information (17, 18). Gathering detailed data on coercive measures inside and outside the hospital at a national level is currently not mandatory and thus not enforced by law. As a consequence, only a small number of hospitals still collect routine data on coercion at present (5, 19, 20). However, such a nationwide overview would be important in order to better examine and understand trends of reducing seclusion followed by periods of indifferent findings.

During the first year of the new legislation, the trend regarding seclusion was more than clear regarding the mental healthcare provider we examined. As data only cover 1 year, we do not know whether the unambiguous numbers of 2020 are going to be sustained. However, medication is now generally seen as treatment in Dutch psychiatric practice, whereas seclusion is increasingly seen as a security measure owing to the way that ward staff approach complex patients in the absence of alternatives (31). This would indicate that the new legislation helped to speed up a development that was slowly gathering pace anyway. It is in keeping with the original ambition of the legislators (1, 4, 32–34) to design legislation focused on treatment.

To examine the hypothesis that the new legislation may have functioned as a catalyst for a focus on treatment, changes in both inpatient and outpatient treatments should be examined over a larger number of institutes and over a number of years, now that the new law has been implemented. One expectation of the new legislation was that intensifying outpatient treatment could prevent admissions. However, the data for 2020 suggest that involuntary outpatient medication rarely happened. It is difficult to say how much perceived and real restrictions during the COVID-19 pandemic may have played a role. The inpatient change, however, is clear. More patients receive involuntary medication, and fewer are subjected to seclusion over far fewer hours. We have to keep in mind that these are only findings from a single year. The expected trend of fewer and shorter admissions after the introduction of the new law cannot be confirmed nor rejected with the limited amount of available data available so far.

However, despite the limited time frame for data collection since the implementation of the new legislation, we have clearly seen a positive trend in keeping with government and patient priorities to focus on treatment and reduce seclusion use. While the reduction of seclusion has been significant from a high baseline internationally with far fewer seclusion hours and fewer patients affected, the increase of enforced-medication use has been significant but remains low by international comparison. In addition, the number of patients being subjected to any type of coercion has dropped and is now in the region of 6%, which is comparable with that in other European countries.

Our findings concern observations at a general level. These need to be supplemented by qualitative research at a departmental level and at the level of patient–staff interaction to understand how and if the implementation of the law has led to a change in the ward culture. Anecdotal evidence from wards suggests that the legislation change encouraged psychiatrists to prescribe treatment more regularly to detained patients, and staff had more time to try and persuade patients to take medication voluntarily because of less staff intense seclusion use. Voluntarily taken medication is, of course, not covered in our dataset of enforced medication. This study is one of the few occasions internationally where the introduction of law seemed to have had an immediate impact on clinicians' behavior. However, qualitative studies are now needed to investigate what may explain the observed change, even though we are yet to discover if the change is sustained over the next years.

### Limitations and Strengths

Several limitations can be identified. The year 2020 was a transition year. On January 1, the new legislation was implemented. The previous legislation was not abruptly terminated. Current treatments were continued in accordance with the remaining legal terms and only transferred to a new treatment after the expiry of previous legal terms. There was therefore a *de facto* coexistence of two legal regimes on the wards for a short period of time. Nevertheless, a clear change was observed.

Another limitation concerns the use of routinely collected data, which may lead to underreporting in an unknown way. We are especially aware of a possible underreporting of outpatient involuntary treatment. Not only are outpatient services reluctant to apply outpatient coercive measures, even though the law allows this, but these services have no experience in recording their measures in a systematic way, which may cause an unknown proportion of unregistered events. As such, we may observe three sources of bias, all due to possible underreporting. First, selection bias could occur in the outpatients and in some inpatients with less overt behavior that is not deemed worthy of reporting. In these patients, registration of involuntary medication could be missed as we observed in previous studies. Furthermore, nurses working in outpatient services may have less knowledge of the requirements of the new law. Second, confirmation bias cannot be ruled out, as the monitoring system was set up to keep track of the main coercive measures, i.e., seclusion and involuntary medication. Less frequently used measures such as mechanical restraint may be missed. Third, with respect to such data in general, we should mention the possibility of publishing bias, as we know from previous studies (17, 21) that our data are favorable compared with other Dutch data.

A third limitation is the use of data from a single hospital. Our communication with other hospitals showed that none of them had yet succeeded in gathering the relevant data in a reliable and valid way. We have therefore started a collaboration with 8 Dutch hospitals. The first findings are expected in 2023, with data collection in 2022. We do not know to which extent the current data are generalizable to other mental health institutes.

A fourth limitation is the COVID-19 pandemic. In a publication by Chow et al. (35) on data of the same Mental Health Trust we collected data from, we observed a decrease in outpatient contacts of patients with psychotic disorders. The number of contacts and the number of patients in care did not change as an effect of COVID-19. The number of patients admitted with COVID-19 to the hospital in 2020 was very limited, with 13 patients only. Instead of increasing pressure on the hospital, the study observed that patients stayed away from care.

A fifth limitation is the extent to which professionals are familiar with the principles of the new law, especially professionals working with outpatients. This may lead to decisions being made that are not entirely in line with the new law. However, this should, if anything, have prevented a trend from developing. Also, we do not know whether informal coercion is applied in the outpatient setting. This may again lead to underreporting of the use of involuntary medication, especially in the outpatient setting. After the implementation of the law, any enforced medication had to be registered by law, but the reliability of this is as yet uncertain. In future studies, the reliability of the data could be improved by cross-checking with the existing prescription software.

A sixth limitation concerns the use of routinely collected data. Even though this collection was done prospectively, these data are subject to missing values. Especially when clinical pressure is high, data registration may be incomplete or not done at the moment of carrying out the measure. For this reason, the data were compared with nurses' and doctors' notes in the medical charts.

A strength is that the examined Mental Health Trust is the first to gather valid data in a reliable way, using checks and balances to validate the findings in the same way since 2012. Another strength is the standardization of the findings, using counters and denominators in a consistent way since 2012. This standardization increases the power of the study as it adds to the sample size and the validity of the time series and regression analyses.

## Conclusions

This study showed a significant decrease in seclusion hours but not in medication events after the introduction of the Dutch Compulsory Care Act (2020). Additional research is important to investigate whether the registered trend is sustainable over time. The expected effect of the new law on the frequency and duration of admissions needs to be investigated in more hospitals and outpatient settings over a longer period of time. In the near future, we hope to extend the current findings to more Mental Health Trusts over more years.

## Data Availability Statement

The datasets presented in this study can be found in online repositories. The names of the repository/repositories and accession number(s) can be found below: Radboud University Repository.

## Author Contributions

SG wrote and designed the study, developed the main questions, drafted the first introduction, results, and discussion sections. He also contributed to the methods section, which was written under the supervision of AW and EN. PL, HH, and GH supervised the writing and design of the introduction, methods results, and discussion sections in an equal way. PL finalized the draft. All authors contributed to the article and approved the submitted version.

## Funding

This study data were based on regular monitoring systems of the Mental Health Care Institute for the purpose of relating data with one another, software was developed by means of a governmental grant provided before the start of the monitoring, Ministry of Health, Welfare and Sport grant (No. 2011 - 5162).

## Conflict of Interest

The authors declare that the research was conducted in the absence of any commercial or financial relationships that could be construed as a potential conflict of interest.

## Publisher's Note

All claims expressed in this article are solely those of the authors and do not necessarily represent those of their affiliated organizations, or those of the publisher, the editors and the reviewers. Any product that may be evaluated in this article, or claim that may be made by its manufacturer, is not guaranteed or endorsed by the publisher.
